# The Effects of SARS-CoV-2 Infection on Female Fertility: A Review of the Literature

**DOI:** 10.3390/ijerph19020984

**Published:** 2022-01-16

**Authors:** Andreea Carp-Veliscu, Claudia Mehedintu, Francesca Frincu, Elvira Bratila, Simona Rasu, Ioana Iordache, Alina Bordea, Mihaela Braga

**Affiliations:** 1 Department of Obstetrics and Gynecology, Carol Davila University of Medicine and Pharmacy, 020021 Bucharest, Romania; andreea_veliscu@yahoo.com (A.C.-V.); francesca.frincu@drd.umfcd.ro (F.F.); elvira.bratila@umfcd.ro (E.B.); ioana.iordache@drd.umfcd.ro (I.I.); alinaelenabordea@yahoo.com (A.B.); 2 Panait Sarbu Clinical Hospital of Obstetrics and Gynaecology, 060251 Bucharest, Romania; simona.rasu8@gmail.com (S.R.); drmihaelabraga@gmail.com (M.B.); 3 Nicolae Malaxa Clinical Hospital Bucharest, 022441 Bucharest, Romania

**Keywords:** SARS-CoV-2, COVID-19, female reproductive system, endometrium, menstrual cycle, ovarian reserve, follicular fluid, oocytes, embryos, in vitro fertilization

## Abstract

As the coronavirus pandemic is far from ending, more questions regarding the female reproductive system, particularly fertility issues, arise. The purpose of this paper is to bring light upon the possible link between COVID-19 and women’s reproductive health. This review emphasizes the effect of SARS-CoV-2 on the hormones, endometrium and menstrual cycle, ovarian reserve, follicular fluid, oocytes, and embryos. The results showed that endometrial samples did not express SARS-CoV-2 RNA. Regarding the menstrual cycle, there is a large range of alterations, but they were all reversible within the following months. The ovarian reserve was not significantly affected in patients recovering from both mild and severe infection in most cases, except one, where the levels of AMH were significantly lower and basal follicle-stimulating hormone (FSH) levels were increased. All COVID-19 recovered patients had positive levels of SARS-CoV-2 IgG in the follicular fluid. The amount of retrieved and mature oocytes and the fertilization rate were unharmed in three studies, except for one study, where the quantity of retrieved and mature oocytes was reduced in patients with higher levels of SARS-CoV-2 antibodies. The numbers of blastocysts, top-quality embryos, and euploid embryos were affected in most of the studies reviewed.

## 1. Introduction

The coronavirus infection broke out in 2019 and has rapidly turned into a global pandemic. It shortly became a healthcare burden, both for the system and for patients. Female fertility concerns arose after abnormal findings in the menstrual cycle: altered menstrual duration, frequency, regularity, and volume (heavier bleeding and clotting), increased dysmenorrhea, and worsened premenstrual syndrome [1]. As the pandemic is far from ending, more and more questions regarding the female reproductive system, especially fertility issues, are arising, and clarifications regarding the possible link between COVID-19 and women’s reproductive health are required.

Coronavirus disease (COVID-19) is thought to be transmitted via direct (deposited on persons) or indirect (deposited on objects) contact [2]. Social distancing is the most effective protective measure, as it is an airborne disease. Manifold transmission ways have been reported: via droplets, surfaces, via inanimate objects, fecal–oral transmission, and biological fluids transmission (saliva, tears), notwithstanding that vertical transmission is also a hypothesis largely debated. Semen transmission presumption cannot be ruled out yet [3], but a recent systematic review concluded that there is no evidence suggesting that COVID-19 is a sexually transmitted disease (STD) [4].

The severe acute respiratory syndrome coronavirus 2 (SARS-CoV-2) encloses a spike protein (S protein) allowing viral binding to the angiotensin-converting enzyme (ACE)2, which acts as a viral receptor and is also widely expressed on the surface of various organs and tissues [5]. In order for the virus to gain entry into the cell and bind to ACE2, cleavage of the S protein is necessary, which is facilitated by the transmembrane serine protease 2 (TMPRSS2). SARS-CoV-2 does not only invade the lungs but also attacks other organs with high ACE2 expression [6], including cardiac, renal, intestinal, and endothelial cells. The testis, ovary, vagina, uterus, and placenta are involved, too [7,8,9,10]. Considering the above, female reproductive may be affected by SARS-CoV-2 infection, as the oocytes and ovarian tissue express medium–high levels of ACE 2 receptor [11,12]. No significant difference has been observed in the ACE2/ transmembrane serine protease 2 (TMPRSS2) expression rate between young and old ovaries and low and high ovarian reserve [13].

Cleaving of the S protein could be achieved by other proteases, which are currently under investigation for increasing SARS-CoV-2 infectivity, such as TMPRSSP4 in the gut epithelial cells [14], cathepsins B and L (CTSB and CTSL, respectively) in TMPRSS2- cells [15]. FURIN in epithelial layers of several mucosal tissues [16,17] and MX dynamin-like GTPase 1 (MX1), which modifies the protein S by neutrophil elastase [18].

Angiotensin II (Ang II) promotes vasoconstriction of the spiral arteries and consequently induces menstruation. The myometrial activity might be influenced by the relationship between Ang II and Ang 1–7 [19]. Therefore, an alteration in the function of Ang II and ACE 2 may determine irregularities of the menstrual cycle, heavy periods, and hyperplastic endometrium [20]. Moreover, high levels of stress have been linked to menstrual cycle changes [21,22]. For this reason, we feel the need to investigate whether COVID-19 infection can induce menstrual cycle alterations, bearing in mind that infertility is already a stress inducer.

ACE 2 has a pivotal role in the ovary: it promotes steroid secretion [23], helps follicle development [24] and oocyte growth [25], influences ovulation [26], and maintains the function of the corpus luteum [27]. Both ACE2 and BSG were found in oocytes, depending on the maturity grade. ACE2 protein was present only in immature oocytes, whereas BSG protein was present in all oocytes, independent of the maturity grade. Potential ways of infecting the oocytes during the IVF process might be: through the blood flow, during the staff handling, or by adding infected semen [28]. Trophectoderm cells of a day-6 embryo have the highest co-expression of the ACE-2 and TMPRSS2 [29,30]. Viotti et al. collected trophectoderm cells from blastocyst-stage embryos donated to research after exposing them to the SARS-CoV-2 virus (infection by spinoculation with GFP-reporter pseudotyped virions) and discovered that the cells from the embryos expressed ACE2 receptor and TMPRSS2 protease are susceptible to the infection through the ACE2 receptor [31].

The oocytes and embryos are therefore susceptible to the SARS-CoV-2 infection. This observation helps draw special attention to ‘in vitro’ (IVF) procedures and embryo transfers. However, it remains unclear whether or not the oocytes and embryos are affected by SARS-CoV-2 infection. This is the main reason why we started this review, in order to draw conclusions that may influence our daily practice [29,30].

Since the actual pandemic caused by the coronavirus disease (COVID-19) is far from coming to an end, we see the importance of talking about the effects on reproductive health. This paper aimed to emphasize the consequences of SARS-CoV-2 infection on the endometrium and menstrual cycle, hormones and ovarian reserve, follicular fluid, oocytes, and embryos.

## 2. Materials and Methods

We conducted a thorough search in the literature published in the PubMed and ScienceDirect databases from the COVID-19 pandemic outbreak (11 March 2020), until 12 December 2021. We used “MeSH” (PubMed) terms “SARS-CoV-2” OR “COVID-19” combined with “female reproductive system” OR “endometrium” OR “menstrual cycle” OR “ovarian reserve” OR “follicular fluid” OR “oocytes” OR “embryos” OR “in Vitro fertilization”. The same terms were searched in free text. We selected 98 articles, and after removing the duplicates, 25 articles remained for the final research in this review. The reference lists of the included studies were also screened for additional literature. Studies in another language than English were excluded.

## 3. Results

### 3.1. The Effects of SARS-CoV-2 on Endometrium and Menstrual Cycle

The endometrium has an important role in embryo implantation. Studies found that it has a low expression of ACE transcript (but no presence of the protein), low expression of TMPRSS4 and Furin, Paired Basic Amino Acid Cleaving Enzyme (FURIN) genes (but medium levels of the proteins), medium expression of Epididymis Secretory Sperm Binding Protein (CTSB), MX Dynamin Like GTPase 1 (MX1), and Basigin (Ok Blood Group) BSG genes (but high levels of the proteins) [32]. The expression of these genes varies through the menstrual cycle. Henarejos et al. published a review in 2020 about the effects of COVID-19 on the endometrium. They concluded that the endometrium should be protected from SARS-CoV-2 infection mediated through TMPRSS2 due to the low presence of ACE2 and medium presence of TMPRSS2. Otherwise, TMPRSS4 appeared significantly increased in the mid-secretory phase, and they found a correlation between TMPRSS4 upregulation and Cathepsin L (CTSL), CTSB, FURIN, MX1. Therefore, the endometrium might be affected by SARS-CoV-2 mediated through TMPRSS4, especially through early and mid-secretory phases. Similar to the activation pathway of ACE2 and TMPRSS2, BSG was an alternative receptor, which modified its expression during the menstrual cycle. They observed that BSG had powerful activation with FURIN, which has a role in the cleavage of the S protein. The high expression of BSG might explain the SARS-CoV-2 infection through other mechanisms, independently of ACE2 [33].

Gomez et al. enrolled 15 women in a study, who were hospitalized for COVID-19, in different phases of the menstrual cycle. They collected endometrial biopsies and tested the samples. All samples tested negative for SARS-CoV-2 RNA, and 10 out of 14 expressed ACE2 receptors [34].

We structured a table with the most notable articles regarding menstrual cycle alterations found during our search (Table 1 and Table 2).

### 3.2. The Effects of SARS-CoV-2 on Hormones and Ovarian Reserve

In the study published by Li et al., Anti Mullerian Hormone (AMH) concentrations were compared in 91 women with COVID-19 and the control group and showed no differences. In the control group, blood samples were taken at any time in the first 4 days of the menstrual cycle, and the case group blood samples were taken at any time in the first 5 days of the menstrual cycle during hospitalization. The results of the sex hormone levels (estrogen, progesterone, testosterone, LH, FSH) were not significantly different between the case and control groups. However, some women had higher concentrations of FSH and LH in the early follicular phase, which could show suppression of ovarian function. As the menstrual cycle disturbances were only transitory, the level of these hormones was not reevaluated. When correlating the serum concentrations results, the study showed little or no impact on ovarian reserve and female fertility [44].

Wang et al. published similar results regarding the levels of FSH, AMH, and antral follicle count (AFC). They enrolled in case group 65 women with positive SARS-CoV-2 IgG undergoing IVF procedures and 195 women in the control group. The levels of FSH and AMH were measured during day 2 or 3 of menstruation and AFC was calculated by transvaginal ultrasound. They observed no differences between women with IgG for SARS-CoV-2 and the control group [46].

Kolanska et al. studied the AMH of 118 women undergoing IVF (with a prior detected level of AMH). The AMH levels of the 14 women who tested positive for SARS-CoV-2 infection and the control group were similar. All women had a history of mild COVID-19 disease [47]. Another study investigated the AMH levels of hospitalized women and still found no differences [44].

Bentov et al. compared the blood and follicular fluid of three groups of women (vaccinated, recovering from COVID infection, and non-vaccinated) to evaluate steroidogenesis. Estradiol and progesterone levels were serologically measured on the trigger day and determined in serum and follicular fluid on the retrieval day. The serum level of progesterone on the day of the trigger was lower in unexposed women, but the level measured on the retrieval day both in blood and follicular fluid was not different. Estradiol level was similar in both groups [48].

In 1132 patients undergoing IVF procedures between April and September 2020 (compared to 997 patients with pre-pandemic procedures), the FSH level was higher (the majority of women had the FSH in the upper two quartiles) at the start of the cycle compared to the levels before the pandemic [49]. An increased level of FSH has been associated with lower pregnancy rates. A study made by Ding et al. and published in March 2021 showed different results in regard to the hormonal ovarian status. In total, 78 SARS-CoV-2 positive women were enrolled in this study. Patients with ovarian pathologies or surgeries were excluded. The main findings were: lower AMH levels, increased FSH levels, and higher testosterone and prolactin levels in women in the COVID-19 group, compared to the age-matched control group. AMH is one of the most accurate markers for ovarian reserve. The findings of these study show that SARS-CoV-2 infection may affect the ovarian reserve. Overall, 48% of the patients in this study experienced in that period mental disorders (anxiety, depression, and sleep disturbances), which may influence the level of prolactin. [45].

### 3.3. The Effects of SARS-CoV-2 on Follicular Fluid

Barragan et al. studied 16 oocytes collected from two asymptomatic positive SARS-CoV-2 women at the time of the egg retrieval. All oocytes were tested for the presence of SARS-CoV-2 RNA, and the result was negative [50]. Data from a case report with a SARS-CoV-2 woman undergoing egg retrieval were published in February 2021 and sustained the findings of Barragan. They reported no presence of SARS-CoV-2 RNA in the follicular fluid. They performed the oocyte retrieval under the paracervical block to reduce the aerosolization of the viral particles, wearing personal protective equipment (PPE). The laboratory staff enforced good laboratory practice (GLP) and the airflow was switched off both in the operating room and the IVF laboratory [51].

The study of Herrero et al. found anti-SARS-CoV-2 Ig G in the follicular fluid of all women undergoing IVF post-COVID-19 and low levels of Vascular endothelial growth factor (VEGF) and IL-1β. The study of Herrero et al. found anti-SARS-CoV-2 Ig G in the follicular fluid of all women undergoing IVF post-COVID-19 infection [52]. The composition of follicular fluid reflects oocyte quality [53,54], and this alteration can have a detrimental effect on reproductive function. Additionally, the study found low levels of vascular endothelial growth factor (VEGF), which could negatively influence the development of ovarian vasculature, decrease the supply of nutrients for the follicles, and thus lead to poor oocyte quality. Moreover, a reduced level of cytokine IL-1β level, which regulates folliculogenesis and atresia [55,56], could negatively impact oocyte quality [52].

Bentov et al. looked at the specific anti-SARS-CoV2 spike protein RBD (receptor binding domain) IgG in serum and the follicular fluid of vaccinated and infected women. They discovered that patients with positive serum levels of anti-COVID IgG also had levels of antibodies in the follicular fluid, and the results were similar between the two groups: infected and vaccinated. Anti-SARS-CoV-2 IgG after vaccination was present in the serum and follicular fluid starting with day 13 after the first dose [48].

### 3.4. The Effects of SARS-CoV-2 on Oocytes and Embryos

A recent cohort study published by Bentov et al. in July 2021 compared three groups of women undergoing egg retrieval: 9 vaccinated, 9 COVID-19 recovered, and 14 non-vaccinated. They evaluated the number of retrieved oocytes, oocyte yield (representing the proportion of retrieved oocytes from the mature follicles seen at the ultrasound on the trigger day), number of mature oocytes, and oocyte quality biomarkers. There were no significant differences between the three groups. To study oocyte quality, investigators measured Heparan Sulfate Proteoglycans (HSPG2) in the follicular fluid, and they detected unchanged levels between the exposed and non-exposed women [48]. The granulosa cells secrete HSPG2 into the follicular fluid and mirror the quantity of estrogen produced under gonadotrophin influence [57].

Wang et al. published a study conducted in the largest IVF center in Wuhan in July 2021, which demonstrated the impact of SAR-CoV-2 infection on women’s fertility. They included women undergoing IVF procedures, negative for SARS-CoV-2 RNA and positive for serum SARS-CoV-2 antibodies (group case), and they compared them to unaffected women by SARS-CoV-2. After propensity score matching, they analyzed 260 women (195 in group control and 65 in group case). They compared the number of the retrieved oocytes, the mature oocyte rate, the fertilization rate, and the blastocyst formation rate, but only the latter was significantly lower (*p* = 0.02). There was no difference between results in the biochemical pregnancy rate, early miscarriage rate, and clinical pregnancy rate [46].

Herrero et al. also studied the IVF outcomes of 46 women who recovered from COVID-19 infection but had different results: a significantly lower number of retrieved and mature oocytes in women with higher SARS-CoV-2 IgG levels [52].

Another study conducted by Orvieto et al. evaluated the IVF outcomes of nine couples before and after COVID-19 infection. While the results of the number of oocytes obtained and fertilization rate were similar, the number of top-quality embryos (TQE) was significantly lower. TQE was considered an embryo with more than seven blastomeres on day 3, ≤10% fragmentation, and blastomeres of equal size [58].

Chamani et al. compared the IVF outcomes of 1881 women who underwent procedures between January and July 2020 to the control group, who underwent procedures in 2019. The mean number of euploid embryos per patient was significantly lower in May and June 2020, and the mean number of blastocysts per patient was significantly higher in April 2020 [59].

In Table 3 we summarized the most important effects of SARS-CoV-2 on oocytes and embryos found in the studies reviewed (Table 3).

## 4. Discussion

In the first stages of the SARS-CoV-2 pandemic, the American Society of Reproductive Medicine (ASRM) and the European Society of Reproductive Medicine (ESHRE) recommended postponing human assisted reproductive technology (ART) treatments, apart from the most urgent cases. Once the spread of the virus became controlled, the societies advised resuming all ART treatments [60].

Nonetheless, this pandemic affected the psychological state of couples, already under the burden of infertility [61]. The inability to undergo ART treatments when the fertility clinics closed due to the pandemic altered the couples’ optimism (older women were more affected due to poor ovarian reserve). Moreover, the distrust about SARS-CoV-2 vaccines put a skeptical light on its fertility implications [62]. Until now, studies showed no negative impact of the vaccine on male fertility but a possible detrimental effect of the COVID-19 infection itself. The high temperature during the COVID-19 infection may be one possible explanation as the effects might correlate with the severity of the infection [63].

ACE2 has an essential role in the endometrium, as it influences spiral artery vasoconstriction, cell proliferation, and the renewal of cells [19,64]. Considering that endometrium renews with every menstruation, the consequences of the virus might be less important. A study showed no SARS-CoV-2 RNA in the endometrial biopsies of positive women [34]. As the viral gene expression increases with age, which might signify that elderly women undergoing ART treatments are at higher risk of viral infection. Therefore, infertility specialists should be more careful with these women [33].

We need a broader understanding of how the menstrual cycle might be influenced by COVID-19 infection, which mechanisms are involved, and the culprit of these changes. The endometrium has a crucial impact on human-assisted reproductive techniques, as responsible for implantation. Another aspect regarding the menstrual cycle is that it is easily influenced by stress. Hence, given the rising stress levels caused by the actual pandemic, we should consider that any type of menstrual abnormalities might occur. The COVID-19 pandemic can be a stress inducer, as war and starvation are [21,22]. In a study from 1961, women before execution stopped menstruating [65]. Therefore, it is universally accepted that extremely stressful experiences induce menstrual cycle changes. Many of the studies included in this review reinforce the idea that high-stress levels were more likely to markup menstrual changes [35,36,40,41]. Long-term stress might also be a contributor. Abnormalities of the menstrual cycle tend to appear more in women >45 years old, probably due to their hypersensitivity of the gonadal hormonal axis around the perimenopausal time [42,66].

As in the means of female fertility, Li et al. concluded that there is no impact on reproductivity, and only strenuous research will give a final resolution on this aspect. The utmost findings included menstrual volume changes, mainly a deceased volume, prolonged menstrual cycle, altered menstruation onset, worsened premenstrual symptoms, missed periods, and reduced libido. Moreover, statistical analysis indicated no significant differences between mild and severe patients in menstrual volume changes. In contrast, significant differences were found regarding the menstrual length [44]. On the other hand, Ding et al. found an increased flow in the more severe group [45]. We need to keep in mind that the hospitalization itself can be a stressful situation that can induce menstrual abnormalities, and only 5.8% of women SARS-CoV-2 positive are admitted (especially those with comorbidities). It is difficult to conclude on this aspect, and more studies are needed [67].

Regarding hormones and ovarian reserve, several studies showed that SARS-CoV-2 has no impact on serum AMH and HSPG2 in follicular fluid. They monitored AMH in recovered patients both from mild COVID-19 infection and from severe disease (hospitalized patients) [44,46,47,48]. The FSH level was higher in patients at the start of the IVF cycle during the pandemic, compared to patients before it, in two of the studies reviewed [44,49]. Steroidogenesis was investigated by serum and follicular fluid estradiol and progesterone levels. One study on 78 women showed lower AMH, higher FSH levels, and higher prolactin and testosterone in COVID-19 patients [45]. Therefore, a potential detrimental effect of SARS-CoV-2 exists. The impairment of the ovarian reserve may appear in hospitalized women due to the systemic inflammatory reaction, but more studies are needed.

Several studies demonstrated that SARS-CoV-2 has no apparent effect on oocytes and embryos, concluding that doctors could safely perform IVF. The available information regarding the absence of SARS-CoV-2 RNA in the follicular fluid of positive women is deficient, but consistent [50,51]. Regarding the presence of SARS-CoV-2 IgG in the follicular fluid of women recovering from COVID-19, all reviewed studies found positive levels [48,50,52]. The study of Herrero et al. detected low VEGF and IL-1β levels in the follicular fluid [52]. In 2019, Mendoza et al. demonstrated better IVF outcomes in patients with higher levels of IL-1β [68]. VEGF has wide implications in ovarian vasculature and oocyte quality [69]. These theories could explain the findings of Herrero—the lower number of retrieved and mature oocytes in patients with higher levels of SARS-CoV-2 antibodies. This study analyzed patients only 3–9 months after COVID-19 infection [52]. More studies are needed to establish if these ovarian alterations are reversible in time.

Many studies have demonstrated how SARS-CoV-2 infection increases oxidative stress [70,71]. Existent studies show the detrimental impact oxidative stress has on the quality of oocytes and embryos, implying that SARS-CoV-2 could alter female fertility [72,73,74]. In this regard, the obtained results by Wang et al. show a significantly lower rate of blastocyst formation, despite the similar number of oocytes, fertilization, implantation, abortion, and clinical pregnancy rate [46]. Similarly, Orvieto et al. found a low TQE rate in 9 couples undergoing IVF procedures in their clinic before and after COVID-19 infection, but they evaluated day-3 embryos. As the procedures were conducted between 8 and 92 days post-infection, taking into consideration the results, the authors recommended postponing the IVF procedures for three months [58]. In concordance with these findings, Chamani et al. found a significantly lower mean number of euploid embryos per patient [59].

We found several biases among the reviewed studies, including data collection, lack of standardized definitions for the parameters researched, and inconsistent data. A big challenge was to find relevant articles with brief results. This would also simplify interpretation and conclusion making. Further investigation is needed to postulate a correct answer to all the questions regarding the female reproductive system and COVID-19 infection.

In conclusion, the results of this review show significant menstrual alterations but reversible and slightly modified ovarian reserve and hormonal balance. Regarding fertility, the highest impact of SARS-CoV-2 infection was seen in the reduced number and quality of embryos. Further investigation is needed to assess the potential impact on the live birth rate.

## Figures and Tables

**Table 1:** A summary of the effects of SARS-CoV-2 on menstrual cycle (before and during COVID-19 pandemic) found in the studies reviewed.

Author and Year	Study Design	Population Sample and Selection Criteria	Data Collection	Main Findings
Bruinvels et al. 2021 [35]	Cross-sectional online questionnaire	749 women Inclusion criteria: ≥18 years Nulliparous Physically active With normal menstrual cycles before COVID-19 At least 9 menstruations or withdrawal bleedings before the pandemic	For a period of 21 days (27 May–17 June 2020)	25%—an increased cycle length 20%—a decreased cycle length >50% had more psychosocial menstrual symptoms: mood changes, reduced focus time and lack of motivation 17% had felt stressed about their menstrual cycle alterations
Malloy et al. 2021 [36]	Cross-sectional online questionnaire	12,302 women	For a period of 13 months (March 2020–April 2021) Through the mobile application Ovia Health’s Fertility	87%—disruptions in cycle pattern 29%—more symptoms during menstruation (abdominal pain, back pain, discharge changes) 27%—increased bleedings A higher level of stress was found in women with menstrual disturbances
Phelan et al. 2021 [37]	Cross-sectional online questionnaire	1031 women Women of child-bearing age Exclusion criteria: Pregnancy Amenorrhoea for any reason	Recruited via social media	No modification with regard to the average menstrual cycle length or total bleeding days 18% experienced heavier bleeding 30% experienced new pain 9% new absence of period (and had none before) 21% experienced more absence of period (and had occasional absence of period before) 53% had increased premenstrual symptoms (PMS) Anxiety and higher level of stress enhanced the incidence of menstrual symptoms
Khan et al. 2021 [38]	Prospective cohort study	127 women Inclusion criteria: - SARS-CoV-2 positive cases - 18–45 years old Exclusion criteria - current pregnancy - recent pregnancy (after 01.2020)	Data extracted from ARIZONA CoVHORT study	16% reported alterations of menstrual pattern, had more COVID-19 symptoms and more likely to be of Hispanic background Out of these, 60% reported irregular periods, 45% increased PMS, 35% had increased cycle length
Takmaz et al. 2021 [39]	Cross-sectional online questionnaire	952 women Inclusion criteria: - women aged 18–40 - with regular periods >1 year before pandemic Exclusion criteria: - pregnancy, postpartum period, or lactation - on hormonal pills or other medication that could influence menstrual cycle - IUD - bleeding problems, thyroid disease, high prolactin, chronic renal insufficiency, cancer - surgical removal of the uterus ± ovaries - major psychiatric pathologies	November 2020– December 2020 Healthcare workers in Turkey	28.7% experienced irregularities of menstrual cycle 10.7% had shorter or longer menstrual cycles 12.9% had the period length changed by more than 9 days 5.8% experienced longer menses 6.5% had intermenstrual spotting Women who experienced irregular periods had significantly higher levels of depression, anxiety, and stress
Demir et al. 2021 [40]	Cross-sectional online questionnaire	263 women Inclusion criteria: - aged 18–45 - with regular menses 6 months before the pandemic Exclusion criteria: - age <18 - with irregular menses - women at menopause - use of oral contraceptives	May 2020 Recruited via social media	Menstrual cycle length and volume decreased significantly No difference was found regarding: - the onset of the period - the grade of dysmenorrhea - the use of analgesics during menstruation Higher levels of stress were detected during the COVID-19 pandemic
Ozimek et al. 2021 [41]	Online survey	210 women Inclusion criteria: - biologically females - aged 18–45 - residents of the United States Exclusion criteria: - use of oral contraceptives - use of hormonal medication - history of gynecological disorders or surgeries - history of pregnancy or lactation in the last 12 months	July–August 2020	54% experienced changes in their periods. Out of these: 50% had prolonged menstrual cycle 34% had a change in the length of the period 50% had modifications of PMS Women with higher levels of stress, had an increased period length and flow
Nguyen et al. 2021 [42]	Retrospective cohort study	18.076 women Inclusion criteria - women who signed up for contraception reasons Exclusion criteria: - lactation - pregnancy in the last year - oral hormonal therapy - diseases that can influence menstrual cycle pattern	March–September 2019 (before pandemic) March–September 2020 (during pandemic) Recruited through Natural cycles mobile application	61.1% felt tremendously stressed during pandemic (46.2% before pandemic) No significant change in the length of the period and the menstrual cycle Only women >45 years were more likely to experience alterations of menstrual cycle (anovulatory cycles, modified length)
Yuksel et al. 2020 [43]	Observational study	58 women Inclusion criteria: - >18 years old - married Exclusion criteria: - menopause - urinary incontinence - gynecological operations - heart or renal problems - hepatitis B, C or HIV - positive test for COVID-19/ living with a COVID-19 positive person	March–April 2020 Recruited via telephone	More women experienced menstrual modifications during the pandemic (27.6 vs. 12.1%)

**Table 2:** A summary of the effects of SARS-CoV-2 on menstrual cycle in COVID-19 positive patients found in the reviewed studies.

Li et al. 2021 [44]	Retrospective cross-sectional study	177 women Inclusion criteria: Women of child-bearing age COVID-19 infection (PCR positive test) Exclusion criteria: - pregnancy - lactating women - gonadal disfunction - previous hysterectomy - previous oophorectomy	June 2019–March 2020 Women admitted in Wuhan hospital	25% had modified menstrual flow (mainly decreased flow) 28% had changes in menstrual cycle pattern (mainly longer cycle) Menstrual abnormalities were mainly found in patients with systemic complications Severely ill patients experienced longer menstrual cycles Follow up showed every patient, except one, returned to their normal cycle
Ding et al. 2021 [45]	Cross-sectional study	78 women (61 mild cases, 17 severe cases) Inclusion criteria: - Positive infection with SARS-CoV-2 - aged <50 Exclusion criteria: - ovarian pathologies or operations - refusal of blood collection - pregnancy - hormonal therapy in the last 3 months	January–March 2020	The more severe cases experienced: - increased dysmenorrhea - irregular menstruations - more frequent amenorrhea - higher flow

**Table 3:** A summary of the effects of SARS-CoV-2 on oocytes and embryos in the studies reviewed.

Bentov et al. [48]	Cohort study	9 women vaccinated 9 COVID-19 recovered 14 non-vaccinated	2 January–3 October 2021	No significant difference among the three groups regarding: - number of retrieved oocytes - oocyte yield - number of mature oocytes
Wang et al. [46]	Retrospective cohort study	195 women in case group - with SARS-CoV-2 IgG - negative for SARS-CoV-2 RNA 65 women in control group	May 2020–February 2021 Reproductive Medicine Center, Tongji Hospital, Wuhan	No significant difference in: - number of the retrieved oocytes - mature oocyte rate - fertilization rate Significantly lower blastocyst formation rate
Herrero et al. [52]	Cohort study	46 women who recovered from COVID-19 infection 34 women that never tested positive for COVID-19	November 2020–April 2021 PREGNA Medicina Reproductiva IVI Buenos Aires InVitro	Significantly decreased in women with higher SARS-CoV-2 infection: - number of retrieved oocytes - oocyte maturity rate
Orvieto et al. [58]	Observational study	9 women undergoing IVF before and after COVID-19 infection and reached ovum pick-up stage	A tertiary, university-affiliated medical center	No significant difference in: - number of oocytes obtained - fertilization rate Significantly lower TQE (top-quality embryos) rate
Chamani et al. [59]	Retrospective cohort study	1881 women undergoing IVF procedures during the pandemic compared to women undergoing IVF procedures in the prior year	6-month period January 2020–June 2020 January 2019–June 2019 NYU Fertility Center	No difference in: - oocyte retrieval rate - mature oocyte rate - fertilization rate Negatively impact on: - the rate of euploid embryos per patient in 05–06.2020

## Data Availability

Not applicable.
